# Association Between Immunohistochemical Profile and Radiographic Presentation of Breast Cancer Skeletal Metastases

**DOI:** 10.3390/diagnostics16020281

**Published:** 2026-01-16

**Authors:** Stanislav Rajkovic, Lazar Miceta, Bojan Petrovic, Nikola Bogosavljevic, Nemanja Jovanovic, Goran Djuricic, Ljubica Simic, Jelena Sopta, Danilo Jeremic

**Affiliations:** 1 Institute for Orthopaedics “Banjica”, Medical Faculty, University of Belgrade, 11000 Belgrade, Serbia; lmiceta@yahoo.com (L.M.); bprobin86@gmail.com (B.P.); boga19@gmail.com (N.B.); nemanjahull@gmail.com (N.J.); 2 University Children’s Hospital, Medical Faculty, University of Belgrade, 11000 Belgrade, Serbia; gorandjuricic@gmail.com; 3 Institute for Pathology, Medical Faculty, University of Belgrade, 11000 Belgrade, Serbia; ljubica.simic87@gmail.com (L.S.); jslabic@yahoo.com (J.S.)

**Keywords:** skeletal metastasis, breast cancer, hormone receptor, HER2, Ki67

## Abstract

**Background/Objectives**: Understanding the biological factors that drive the behavior and clinical presentation of breast cancer (BC) skeletal metastases (SM) is critical for improving diagnostic accuracy and guiding treatment strategies. However, evidence regarding the immunohistochemical (IHC) profiles of SM and their association with radiographic characteristics and clinical features remains limited. This study aimed to evaluate the relationship between estrogen receptor (ER), progesterone receptor (PR), HER2 receptor status, and Ki-67 proliferation index with the radiographic presentation of SM in patients with BC. **Methods**: A total of 185 SM samples from individual BC patients were analyzed. IHC expressions of ER, PR, HER2, and Ki-67 were determined for each sample. Clinical and radiological data were retrieved from medical records. IHC profiles were compared between metastases demonstrating purely lytic versus mixed radiographic patterns. **Results**: Of the 185 cases, 66 exhibited a lytic pattern, and 119 demonstrated a mixed pattern. Lytic metastases showed a significantly higher rate of HER2 positivity compared with mixed lesions. The Ki-67 index was also significantly higher in lytic metastases, with a cutoff value of 25 yielding a sensitivity of 92.98% and specificity of 89.84% (positive likelihood ratio 9.16; negative likelihood ratio 0.08). No significant differences were observed between groups in ER or PR expression. **Conclusions**: SM from BC with a lytic radiographic presentation are more likely to exhibit HER2 positivity and a Ki-67 index above 25. Assessing the IHC status of SM may help identify patients at elevated risk for skeletal complications, including pathological fracture, and may support more tailored surgical and systemic treatment planning.

## 1. Introduction

Breast cancer (BC) is the most common malignancy affecting women worldwide, with incidence rates ranging from 33 per 100,000 in China to 105 per 100,000 in France [1]. Despite substantial advances in diagnosis and treatment, BC continues to cause more than 660,000 deaths annually, with the highest mortality observed in low-resource regions. In 2022, mortality rates reached 12.2 per 100,000 in the United States and 13–16 per 100,000 across Western Europe [2].

Advances in diagnosis and treatment have improved survival in breast cancer (BC); however, skeletal metastases (SM) remain a major source of morbidity and mortality. Early dissemination to bone is common, with SM reported in 65–75% of patients, making bone involvement a significant contributor to BC-related deaths [3]. Accurate identification of skeletal metastases, most often via plain radiography or computed tomography (CT) [4], is essential for evaluating disease progression and anticipating skeletal complications. Radiographic assessment enables classification of lesions as lytic, blastic, or mixed, a distinction with important clinical implications [5,6]. Unlike most solid tumors, which predominantly produce lytic bone lesions, BC frequently gives rise to mixed-pattern metastases at rates similar to those of purely lytic lesions [7].

Pathological fracture is a severe and debilitating consequence of SM, emphasizing the need to better understand factors that influence metastatic behavior within bone. Clinical risk assessment tools, such as Mirel’s score for long bones and the Spinal Instability Neoplastic Score (SINS) for vertebrae, rely on radiographic evaluation, including lesion type (lytic, mixed, or blastic), as a key parameter, reflecting the varying fracture risk associated with each pattern [5,6]. Beyond radiologic appearance, tumor biology plays a significant role in disease progression. Expression of estrogen receptor (ER), progesterone receptor (PR), human epidermal growth factor receptor-2 (HER2), and the Ki-67 proliferation index [8,9] are established prognostic and predictive markers in primary BC. Their expression patterns correlate with tumor aggressiveness, metastatic potential, and overall survival [10,11]. These associations raise the possibility that immunohistochemical (IHC) characteristics may also influence the radiographic phenotype and clinical behavior of skeletal metastases [11,12].

While most previous studies have focused on hormone receptor status in primary BC and, to a lesser extent, in metastatic disease, the relationship between IHC profiles and the radiographic appearance of SM has not been specifically investigated. This study aims to address this gap by evaluating ER, PR, HER2, and Ki-67 expression in BC skeletal metastases exhibiting either purely lytic or mixed radiological patterns, in order to assess their potential influence on metastatic behavior within bone. In addition, demographic and clinical factors were examined for possible associations with SM radiographic presentation.

## 2. Materials and Methods

### 2.1. Clinical Data

This retrospective observational study systematically analyzed clinical, radiological, and pathohistological data from patients diagnosed with SM secondary to BC. A total of 185 female patients with biopsy-confirmed metastatic BC, treated between 29 March 2011 and 11 March 2024 at a regional reference center specializing in bone and soft tissue oncology, were included in the study. Male patients, those who underwent surgical intervention at external institutions, and individuals with incomplete medical records were excluded. Additionally, only SM with fully characterized ER, PR, HER2, and Ki-67 status were included in the analysis. The patients’ clinical, radiological and pathological data were accessed between 1 April 2024 and 31 August 2024.

Clinical data were retrieved from patient medical records. Radiographic assessment of SM was performed using plain radiography and/or CT. Lesions were classified as pure lytic if no sclerotic zone was observed and as mixed if sclerotic zones were present—this in all cases, was a mix of almost equal ratios of improperly distributed lytic and sclerotic zones. All radiographies and CT scans were analyzed independently by a radiologist specialized in skeletal oncology and an experienced orthopedic oncology surgeon. In almost all cases, the opinions were matched, and in rare cases of initial disagreement, these two specialists met to discuss and found consensus.

Biopsy procedures were conducted according to clinical indication. Bone lesions associated with pathological fractures were routinely biopsied at the fracture site, typically during surgical fracture stabilization. Lesions without fractures were biopsied when the primary tumor origin was uncertain or when a substantial interval had elapsed since the initial BC diagnosis, raising suspicion for a second malignancy. In patients with multiple SM, the most accessible lesion was selected for biopsy to optimize procedural feasibility.

### 2.2. Ethical Approval and Consent to Participate

The study was approved by the Ethical Committee of the Medical Faculty, University of Belgrade, number 1322/V-3 and the Ethical Committee of the Institute for Orthopaedics Banjica, number I-58/9, and the research was carried out in compliance with 1964. Declaration of Helsinki. It was conducted retrospectively as data analysis of an existing data bank without any additional experiment on human or animal tissue. All data were fully anonymized. Written informed consent to participate, including permission to use medical record data for research purposes, was obtained from all participants.

### 2.3. Immunohistochemistry

The immunohistochemical profile, including ER, PR, HER2, and the Ki-67 proliferative index, was systematically evaluated. Serial sections (5 µm thick) were prepared for immunohistochemical evaluation using U.S. Food and Drug Administration (FDA)-approved primary rabbit monoclonal antibodies (Ventana Medical Systems, Oro Valley, AZ, USA), specifically PR (1E2 clone), ER (6F11 clone), HER2 (4B5 clone), and Ki-67 (M7240 Clone MIB-1, diluted 1:100; Dako). Appropriate positive and negative controls were included for each specimen.

Slide evaluation was performed in accordance with the American Society of Clinical Oncology (ASCO) and College of American Pathologists (CAP) guidelines [13,14]. ER and PR expression was assessed based on the proportion of positively stained tumor cells and staining intensity, resulting in a total score ranging from 0 to 8. The percentage of stained tumor cells was categorized as follows: 0 (negative), 1 (<1%), 2 (1–10%), 3 (11–33%), 4 (34–66%), and 5 (67–100%). Staining intensity in the predominant tumor area was graded as 0 (none), 1 (weak), 2 (moderate), or 3 (strong).

HER2 expression was assessed based on staining intensity and the proportion of positively stained cells, classified as negative (score 0 or 1+), equivocal (score 2+), or positive (score 3+). Equivocal cases underwent repeated immunohistochemical testing, and if results remained inconclusive, fluorescence in situ hybridization (FISH) was performed to determine HER2 status.

The Ki-67 proliferative index was determined by quantifying tumor cells exhibiting nuclear staining and reported as a percentage of the total tumor cell population.

### 2.4. Statistical Analysis

Numerical data were presented as mean with a standard deviation and range. Categorical variables were summarized by absolute numbers with percentages. Distribution of numerical data was analyzed using the Kolmogorov–Smirnov test. Chi-squared tests, *t* tests for independent samples and the Mann–Whitney test were used to assess the differences between groups. The odds ratio and 95% Confidence Intervals were also calculated. In all analyses, the significance level was set at 0.05. Statistical analysis was performed using SPSS v.28.0 software (SPSS Inc., Chicago, IL, USA).

## 3. Results

### 3.1. Demographic and Clinical Characteristics

A total of 185 patients were included in the analysis, with a mean age of 61.6 ± 10.5 years (range 33–88). Among them, 127 of 185 patients (68.6%) had a confirmed diagnosis of BC prior to SM biopsy and had undergone previous local treatment (surgery and/or radiotherapy) as well as systemic therapy, including chemotherapy, hormonal therapy, and/or biological therapy. In the remaining 58 patients (31.4%), BC was diagnosed for the first time based on the bone biopsy. Among those with previously established BC, the mean interval from primary diagnosis to SM detection was 79.3 months (range 1–288). None of the examined demographic variables showed a significant association with the radiological presentation of SM (*p* > 0.05) (Table 1).

At the time of SM diagnosis, 100 patients (54.1%) presented with a single skeletal metastasis, while 85 patients (45.9%) had involvement of two or more bones. The spine was the most commonly affected site, with metastases identified in 155 cases (83.8%), followed by the pelvis in 129 cases (69.7%) and the femur in 100 cases (54.1%) (Table 2). Mixed-pattern SM, characterized by combined osteolytic and osteoblastic components, were observed in 119 patients (64.3%), whereas purely lytic lesions were present in 66 patients (35.7%).

Regarding anatomical distribution, none of the individual flat bones analyzed showed a significant difference between lytic and mixed presentations (*p* > 0.05); however, a significant association with mixed presentation of metastasis was demonstrated when all flat bones were analyzed as a group (*p* = 0.046). Within the spine, however, cervical metastases demonstrated a significant association with radiographic pattern, with lytic lesions predominating (83.3% lytic vs. 16.7% mixed; *p* = 0.013). Thoracic and lumbar involvement did not differ significantly between groups (*p* > 0.05).

Among long bones, femoral neck metastases were significantly associated with radiographic appearance (*p* = 0.015), with mixed lesions representing the majority (82.4%). Additionally, tibial and fibular metastases showed a significant difference between groups (*p* = 0.019), with lytic lesions occurring exclusively in this region within the analyzed cohort (Table 2).

### 3.2. Immunohistochemistry

Analysis of hormone receptor expression showed no significant associations between radiographic presentation and PR or ER status. PR positivity was observed in 36 lytic metastases (19.46%) and 54 mixed metastases (29.19%), with no significant difference between groups (*p* = 0.233). Similarly, ER expression did not differ significantly, with ER-positive status present in 49 lytic lesions (26.49%) and 96 mixed lesions (51.89%) (*p* = 0.310).

In contrast, HER2 expression demonstrated a significant association with radiographic pattern. The proportion of HER2-positive metastases was higher in the lytic group (31/66, 47.0%) than in the mixed group (36/119, 30.3%), while HER2-negative metastases were more common in the mixed group (83/119, 69.7%) than in the lytic group (35/66, 53.0%). This association was significant, with lytic lesions showing higher odds of HER2 positivity (*p* = 0.023).

**Figure 1:**
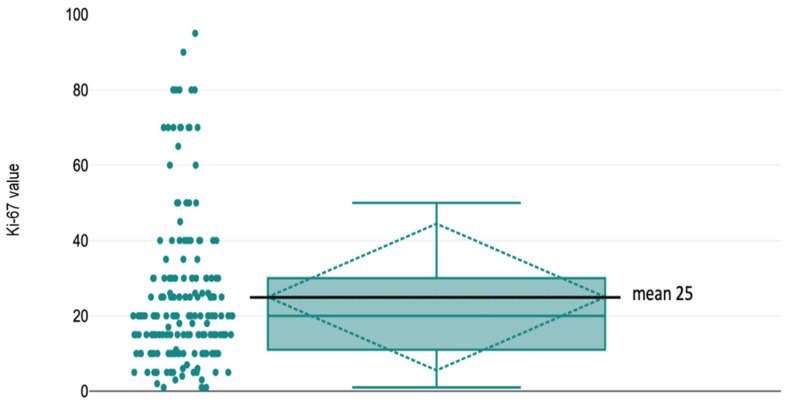
Ki-67 value distribution in whole cohort.

A marked difference was also observed in Ki-67 proliferation index, which was significantly higher in the lytic group (mean 41.65; range 10–90) compared with the mixed group (mean 15.76; range 1–95) (*p* < 0.001). Using a cutoff value of 25%, 13 lytic lesions exceeded this threshold compared with 115 mixed lesions, whereas metastases with Ki-67 ≤25% were far more common in the mixed group (115 cases) than in the lytic group (13 cases). The association was statistically significant (OR = 0.008; 95% CI: 0.003–0.037; *p* < 0.001), indicating that a Ki-67 index >25% strongly correlated with a lytic radiographic pattern (Table 3, Figure 1).

**Figure 2:**
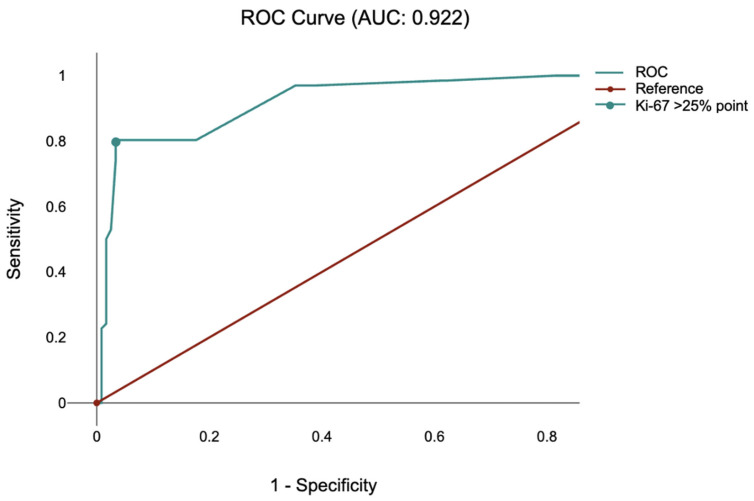
Receiver operating characteristic (ROC) for Ki-67.

Receiver operating characteristic (ROC) analysis was performed to assess the ability of Ki-67 to discriminate lytic from mixed radiographic presentations. Ki-67 showed excellent discriminatory performance (AUC = 0.922) (Figure 2). Using ROC-based threshold selection, the optimal cut-off was Ki-67 > 25% (maximizing the Youden index), yielding a sensitivity of 80.3% and specificity of 96.6%. The corresponding likelihood ratios were LR + 23.89 and LR − 0.20.

**Figure 3:**
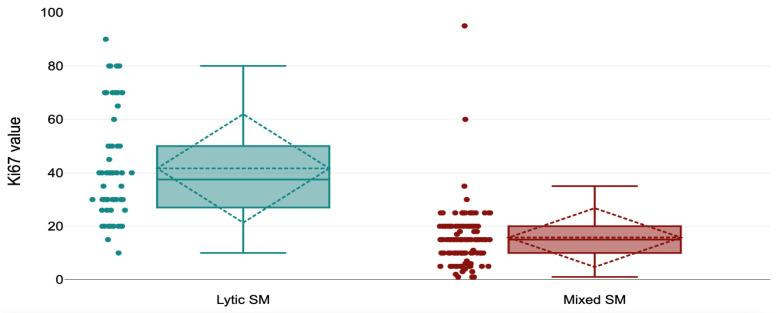
Ki67 distribution by value at lytic and mixed radiographic presentation.

**Figure 4:**
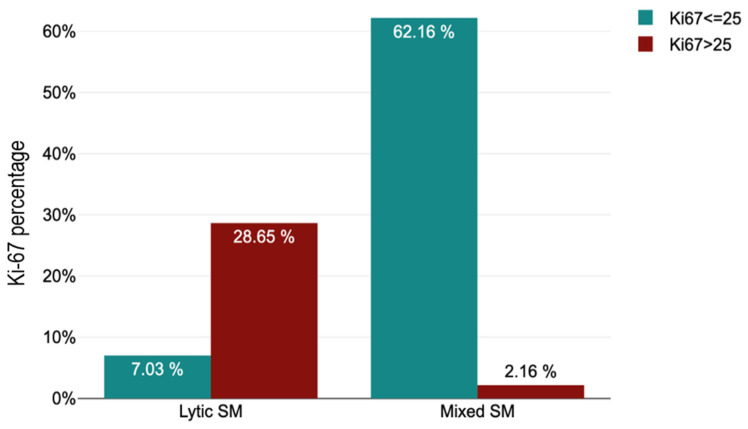
Ki67 distribution by cut-off value at lytic and mixed radiographic presentation.

A Ki-67 value greater than 25% was observed in 53 of 66 lytic SM (80.3%), whereas a Ki-67 value ≤ 25% was present in 115 of 119 mixed SM (96.64%) (Table 3; Figure 3 and Figure 4).

## 4. Discussion

This study demonstrates that HER2 positivity and, in particular, a high Ki-67 proliferation index, are strongly associated with the development of lytic SM in BC. A Ki-67 index greater than 25% and HER2 expression appear to serve as independent markers of a more aggressive, bone-destructive metastatic phenotype, which is clinically relevant given the higher risk of pathological fracture associated with lytic lesions. These findings indicate that the immunohistochemical profile of SM may offer valuable insight into metastatic behavior within bone and can assist clinicians in identifying patients who may benefit from prophylactic surgical stabilization.

The biological divergence between primary tumors and metastatic lesions is well recognized, with discordance in ER, PR, HER2, and Ki-67 expression documented in 18–56% of hormone receptor profiles and 6–48% of HER2 assessments [15,16,17]. As emphasized in the American Society of Clinical Oncology (ASCO) and the College of American Pathologists (CAP) guidelines, treatment decisions should be based on the IHC characteristics of metastatic tissue when feasible, as these markers often differ from those of the primary tumor and may more accurately reflect current tumor biology [16]. Our findings reinforce the value of this approach by showing that metastatic IHC markers, rather than primary tumor markers, are directly linked to radiographic behavior in bone.

A recent study of 1100 breast cancer patients, including 174 with confirmed skeletal metastases, offers additional insight into the biological and clinical factors associated with skeletal involvement [18]. The authors reported that larger primary tumor diameter and a higher number of positive axillary lymph nodes were significantly associated with the development of skeletal metastases, underscoring the importance of tumor burden and nodal spread as drivers of skeletal dissemination. Although correlations were observed between ER and PR expression and between ER and Ki-67 levels, none of the classic immunohistochemical markers, such as ER, PR, HER2, or Ki-67, showed a significant independent association with the presence of skeletal metastases. Rather than predicting the occurrence of skeletal metastases, our study evaluated marker associations with metastatic behavior within bone. Among patients with established skeletal metastases, HER2 positivity and high Ki-67 were associated with a lytic radiographic phenotype. This contrast highlights an important distinction: while traditional tumor markers may not reliably predict which patients will develop skeletal metastases, their expression within established metastatic lesions, particularly HER2 and Ki-67, may have significant implications for metastatic behavior within bone and the associated risk of skeletal complications.

Ki-67 is a nuclear protein expressed exclusively in proliferating cells. During interphase, it is localized within the nucleus, while during mitosis, it relocates to the surface of chromosomes. Owing to this cell-cycle-dependent expression, Ki-67 serves as a well-established marker of cellular proliferation, and the Ki-67 proliferative index reflects the proportion of actively dividing tumor cells [19]. Despite its long-standing recognition, Ki-67 has not been uniformly incorporated into routine BC assessment. This limited clinical integration stems largely from the lack of standardized analytical procedures, uncertainty regarding optimal cutoff thresholds, and insufficient high-level evidence supporting its prognostic and predictive value. Currently, Ki-67 is primarily used to assess primary tumor aggressiveness and to predict response to neoadjuvant therapy. Triple-negative BC typically exhibits the highest Ki-67 indices, consistent with the aggressive biological behavior of this subtype [20]. Furthermore, an elevated Ki-67 index after neoadjuvant therapy is associated with poor therapeutic response and an unfavorable prognosis [21]. While most prior investigations have examined Ki-67 expression in primary BC tissue, the proliferative index within metastatic lesions, and particularly its relationship to skeletal metastatic behavior, has not been well characterized. Notably, Szekely et al. [22] reported that Ki-67 was the only marker demonstrating a significant change specifically in skeletal metastases compared with the primary tumor. This observation supports the notion that skeletal metastases may harbor biological characteristics distinct from metastases in other organs. Such site-specific phenotypic divergence highlights the importance of assessing the immunohistochemical profile directly within bone lesions, as these alterations can influence metastatic behavior, treatment responsiveness, and the likelihood of skeletal complications [23]. Markers such as HER2 and Ki-67 may help identify lesions at increased risk for aggressive bone destruction, thereby assisting clinicians in selecting patients who could benefit from earlier prophylactic stabilization and tailored systemic therapy. Incorporating this biological information into clinical decision-making may improve the ability to anticipate disease progression and optimize individualized patient management.

This study has several limitations, including its retrospective design and the absence of a complete breast cancer cohort for comparison. In addition, interobserver agreement for the lytic versus mixed radiographic classification was not formally quantified, and some degree of classification variability cannot be excluded. Also, as only lesions with a complete immunohistochemical profile were included, selection bias is possible, which may limit the external validity of our findings. Nevertheless, the analysis of a large number of metastatic bone samples offers meaningful conclusions with direct clinical relevance. Given the demonstrated differences between primary tumors and skeletal metastases, routine biopsy of newly diagnosed SM may provide critical information to support personalized treatment strategies.

## 5. Conclusions

This study demonstrates that HER2 positivity and a high Ki-67 proliferation index are strongly associated with a lytic radiographic pattern in SM of BC. In this way, these immunohistochemical features may help identify patients at increased risk of pathological fracture. Given the documented biological differences between primary tumors and metastatic lesions, evaluation of the immunohistochemical profile directly within skeletal metastases provides clinically valuable information that cannot be reliably inferred from the primary tumor alone. Incorporating these metastatic tissue characteristics into clinical decision-making may support earlier prophylactic surgical intervention and more individualized systemic therapy. Further prospective studies are warranted to validate these findings and to clarify the biological mechanisms driving the observed radiographic phenotypes. Also, independence from other factors (e.g., prior therapy, site of metastasis) is to be tested in a multivariable model. Serum bone turnover markers warrant further investigation as potential additional tools for prognostic assessment. Fracture risk and surgical outcomes were not endpoints of this study, and the proposed clinical utility remains hypothetical.

## Figures and Tables

**Table 1:** Demographic and clinical characteristics.

Variable	Radiographic Presentation		* OR, 95% CI	*p*-Value
	Lytic	Mixed		
	(*n* = 66)	(*n* = 119)		
Age (years), mean (range)	62.7 ± 10.9 (36–88)	61.01 ± 10.3 (33–83)		*p* = 0.298
Unknown primary BC at SM diagnosis—No previous therapy	23 (12.43%)	35 (18.92%)	0.779, 0.410–1.479	*p* = 0.446
Known primary BC at SM diagnosis—Previous therapy	43 (23.24%)	84 (45.41%)		
Time from primary BC diagnosis to SM diagnosis (months), mean (range)	68.88 (1–288)	84.61 (1–264)		*p* = 0.142

* Odds ratio (OR) was obtained from logistic regression analysis, with radiographic presentation as the dependent variable coded as mixed (1) and lytic (2).

**Table 2:** Distribution of skeletal metastases and radiological presentation.

			Radiographic Presentation		*p*-Value		
			Lytic	Mixed			
**FLAT**	**Skull**		0 (0%)	1 (100%)	*p* = 0.455		*p* = 0.046
	**Sternum & ribs**		3 (30%)	7 (70%)	*p* = 0.700		
	**Skapula & clavicle**		2 (40%)	3 (60%)	*p* = 0.838		
	**Pelvis**		10 (7.75%)	119 (92.25%)	*p* = 0.068		
	**Sacrum**		1 (50%)	1 (50%)	*p* = 0.671		
**SPINE**	**Cervical**		5 (83.33%)	1 (16.67%)	*p* = 0.013		*p* = 0.214
	**Thoracal**		19 (44.19%)	24 (55.81%)	*p* = 0.184		
	**Lumbal**		23 (21.7%)	83 (78.3%)	*p* = 0.520		
**LONG**	**Humerus**	**Proximal**	4 (36.36%)	7 (63.64%)	*p* = 0.961	*p* = 0.511	*p* = 0.556
		**Diaphysis**	6 (46.15)	7 (53.85%)	*p* = 0.413		
	**Radius & Ulna**		0 (0%)	1 (100%)	*p* = 0.455		
	**Femur**	**Neck**	6 (17.65%)	28 (82.35%)	*p* = 0.015	*p* = 0.576	
		**Trochanteric**	13 (34.21%)	25 (65.79%)	*p* = 0.832		
		**Diaphysis**	12 (42.86%)	16 (57.14%)	*p* = 0.389		
	**Tibia & Fibula**		3 (100%)	0 (0%)	*p* = 0.019		
**Solitary metastasis**			36 (19.46%)	64 (34.59%)	* OR = 0.969 95% CI = 0.530–1.773		*p* = 0.92
**Multiple metastases**			30 (16.22%)	55 (29.73%)			

* Odds ratio (OR) was obtained from logistic regression analysis, with radiographic presentation as the dependent variable coded as mixed (1) and lytic (2).

**Table 3:** Cytopathological characteristics of the breast skeletal metastases.

	Radiographic Presentation				OR, CI 95%	*p*-Value
	Lytic		Mixed			
**Receptor**	**PR**					
**Count**	PR+ 36 (19.46%)	PR− 30 (16.21%)	PR+ 54 (29.19%)	PR− 65 (35.14%)	OR = 1.441 CI 95% = 0.789–2.623	*p* = 0.233
**Receptor**	**ER**					
**Count**	ER+ 49 (26.49%)	ER− 17 (9.19%)	ER+ 96 (51.89%)	ER− 23 (12.43%)	OR = 0.691 CI 95% = 0.338–1.412	*p* = 0.310
**Receptor**	**HER2**					
**Count**	HER2+ 31 (16.76%)	HER2− 35 (18.92%)	HER2+ 36 (19.46%)	HER2− 83 (44.86%)	OR = 2.042 CI 95% = 1.096–3.803	***p*** **= 0.023**
	**Ki67**					
**Mean**	41.65 (Range 10–90)		15.76 (Range 1–95)			***p*** **< 0.001**
**Cut-off value**	≤25	>25	≤25	>25	OR = 0.008 CI 95% = 0.003–0.037	***p*** **< 0.001**
	13 (7.03%)	53 (28.65%)	115 (62.16%)	4 (2.16%)		

The percentages are the proportion of the whole cohort.

## Data Availability

The data that support the findings of this study are available on request from the corresponding author.

## References

[1] Siegel R.L., Miller K.D., Fuchs H.E., Jemal A. (2022). Cancer statistics, 2022. CA Cancer J. Clin..

[2] Heer E., Harper A., Escandor N., Sung H., McCormack V., Fidler-Benaoudia M.M. (2020). Global burden and trends in premenopausal and postmenopausal breast cancer: A population-based study. Lancet Glob. Health.

[3] Zhang H., Zhu W., Biskup E., Yang W., Yang Z., Wang H., Qiu X., Zhang C., Hu G., Hu G. (2018). Incidence, risk factors and prognostic characteristics of bone metastases and skeletal-related events (SREs) in breast cancer patients: A systematic review of the real-world data. J. Bone Oncol..

[4] Łukaszewski B., Naza J., Goch M., Łukaszewska M., Stępiński A., Jurczyk M.U. (2017). Diagnostic methods for detection of bone metastases. Contemp. Oncol..

[5] Mirels H. (1989). Metastatic disease in long bones. A proposed scoring system for diagnosing impending pathologic fractures. Clin. Orthop. Relat. Res..

[6] Fisher C.G., DiPaola C.P., Ryken T.C., Bilsky M., Shaffrey C.I., Berven S.H., Harrop J.S., Fehlings M.G., Boriani S., Chou D. (2010). A Novel Classification System for Spinal Instability in Neoplastic Disease: An Evidence-Based Approach and Expert Consensus From the Spine Oncology Study Group. Spine.

[7] Johnson R.W., Suva L.J. (2018). Hallmarks of Bone Metastasis. Calcif. Tissue Int..

[8] Yang H., Wang R., Zeng F., Zhao J., Peng S., Ma Y., Chen S., Ding S., Zhong L., Guo W. (2020). Impact of molecular subtypes on metastatic behavior and overall survival in patients with metastatic breast cancer: A single-center study combined with a large cohort study based on the Surveillance, Epidemiology and End Results database. Oncol. Lett..

[9] Wu Q., Li J., Zhu S., Wu J., Chen C., Liu Q., Wei W., Zhang Y., Sun S. (2017). Breast cancer subtypes predict the preferential site of distant metastases: A SEER based study. Oncotarget.

[10] Ferguson N.L., Bell J., Heidel R., Lee S., Vanmeter S., Duncan L., Munsey B., Panella T., Orucevic A. (2013). Prognostic value of breast cancer subtypes, Ki-67 proliferation index, age, and pathologic tumor characteristics on breast cancer survival in Caucasian women. Breast J..

[11] Bae S.Y., Kim S., Lee J.H., Lee H.C., Lee S.K., Kil W.H., Kim S.W., Lee J.E., Nam S.J. (2015). Poor prognosis of single hormone receptor- positive breast cancer: Similar outcome as triple-negative breast cancer. BMC Cancer.

[12] Rajkovic S. (2024). Skeletne metastaze karcinoma dojke–uticaj hormonskih i HER2 receptora. Med. Podml..

[13] Hammond M.E., Hayes D.F., Dowsett M., Allred D.C., Hagerty K.L., Badve S., Fitzgibbons P.L., Francis G., Goldstein N.S., Hayes M. (2010). American Society of Clinical Oncology/College of American Pathologists guideline recommendations for immunohistochemical testing of estrogen and progesterone receptors in breast cancer. J. Clin. Oncol..

[14] Wolff A.C., Hammond M.E., Hicks D.G., Dowsett M., McShane L.M., Allison K.H., Allred D.C., Bartlett J.M., Bilous M., Fitzgibbons P. (2013). Recommendations for human epidermal growth factor receptor two testing in breast cancer: American Society of Clinical Oncology/College of American Pathologists clinical practice guideline update. J. Clin. Oncol..

[15] Holdaway I.M., Bowditch J.V. (1983). Variation in receptor status between primary and metastatic breast cancer. Cancer.

[16] Lindström L.S., Karlsson E., Wilking U.M., Johansson U., Hartman J., Lidbrink E.K., Hatschek T., Skoog L., Bergh J. (2012). Clinically used breast cancer markers such as estrogen receptor, progesterone receptor, and human epidermal growth factor receptor 2 are unstable throughout tumor progression. J. Clin. Oncol..

[17] Lindstrom L., Howell S., Astrom G. (2010). Controversies in the management of metastatic breast cancer: Biologic evaluation of breast cancer–should metastases be biopsied?. American Society of Clinical Oncology 2010 Educational Book.

[18] Başdelioğlu K. (2021). Bone metastasis: Evaluation of 1100 patients with breast cancer. Int. J. Clin. Exp. Pathol..

[19] Scholzen T., Gerdes J. (2000). The Ki-67 protein: From the known and the unknown. J. Cell. Physiol..

[20] Dowsett M., Nielsen T.O., A’Hern R., Bartlett J., Coombes C.R., Jack Cuzick J., Ellis M., Henry N.L., Hugh J.C., Lively T. (2011). Assessment of Ki67 in breast cancer: Recommendations from the International Ki67 in Breast Cancer working group. J. Natl. Cancer Inst..

[21] Hashmi A.A., Hashmi K.A., Irfan M., Khan S.M., Edhi M.M., Ali J.P., Hashmi S.K., Asif H., Faridi N., Khan A. (2019). Ki67 index in intrinsic breast cancer subtypes and its association with prognostic parameters. BMC Res. Notes.

[22] Szekely B., Nagy Z.I., Farago Z., Kiss O., Lotz G., Kovacs K.A., Madaras L., Udvarhelyi N., Dank M., Szentmartoni G. (2017). Comparison of immunophenotypes of primary breast carcinomas and multiple corresponding distant metastases: An autopsy study of 25 patients. Clin. Exp. Metastasis.

[23] Rajkovic S., Charalambous M., Charalambous C., Simic L., Djuricic G., Dundjerovic D., Miceta L.D., Milicic B.R., Sopta J.P. (2023). Higher rate of progesterone receptor positivity in skeletal metastases of breast cancer with a pathological fracture vs those without fracture. Int. J. Cancer.

